# Short-term effects of acoustically controlled auditory training in children with impaired school performance

**DOI:** 10.1590/2317-1782/e20240135en

**Published:** 2025-05-26

**Authors:** Júlia Roja Tavoni, Maria Francisca Colella dos Santos

**Affiliations:** 1 Programa de Pós-graduação em Saúde da Criança e do Adolescente, Centro de Investigação em Pediatria, Universidade Estadual de Campinas – UNICAMP - Campinas (SP), Brasil.

**Keywords:** Auditory Perception Disorders, Neuronal Plasticity, Rehabilitation, Child, Speech, Language and Hearing Sciences

## Abstract

**Purpose:**

To investigate the effectiveness of acoustically controlled auditory training (ACAT) and the maintenance of auditory skills acquired by children with impaired school performance.

**Methods:**

Sample consisting of 20 schoolchildren: Control Group (CG – N=10) and Intervention Group (IG – N=10), aged from 8 to 13 years old, results below expectations in a school performance screening, adequate results in a cognitive screening and diagnosed with central auditory processing disorder (CAPD) after a battery of behavioral tests. The CG did not receive any type of intervention, only evaluation and reevaluation after three months. The IG was assessed immediately after eight ACAT sessions and three months post-intervention. Auditory processing assessment and reassessment data were subjected to statistical analysis (p<0.05).

**Results:**

The sample was considered homogeneous regarding gender, age, education level and academic performance. Regarding CAP assessment data, both groups were homogeneous (excepting the Synthetic Sentence Identification – SSI test). Children from IG improved their performance on the Dichotic Digit, Consonant Vowel, SSI and Frequency Pattern (FPT) tests immediately after the intervention. After three months of ACAT, these values remained the same or increased, except for FPT. In the qualitative analysis, between normal and altered, the IG maintained adequate results after three months of ACAT. In the CG, six children maintained altered results and four reached normality in the reassessment after three months.

**Conclusion:**

ACAT proved to be effective for rehabilitating the auditory skills of children with impaired school performance even three months after the end of the intervention.

## INTRODUCTION

The learning process is primarily based on language, since expressive and comprehensive language skills are essential for the beginning of school life. The integrity of the auditory system and skills is also a fundamental part of this process, since it is through them that the child has access to information about the world around them, even before acquiring spoken language.

The development of auditory skills begins in the intrauterine life, and these skills are improved throughout growth, based on sound experiences and neuromaturation of the structures of the central nervous system. Auditory skills allow the individual to understand which sounds are relevant in noisy environments, sustain auditory attention over time, compare, recover, and integrate different sound stimuli^(1)^.

Considering the impact of hearing on language acquisition and the neuromaturation in the first years of life, the maturation and improvement of auditory skills directly affect a child’s social, emotional, and cognitive development^(2)^. At the same time, children with alterations in the auditory system in early childhood are more likely to face obstacles in learning how to read and write and, consequently, in the development of reading and writing skills^(3)^.

The processes and mechanisms of the auditory nervous system have been defined as belonging to central auditory processing (CAP). As sound travels through the auditory pathways, the individual is able to detect, localize, recognize and ultimately interpret the auditory information received^(4)^. Then, CAP is described as a set of skills required for the analysis and interpretation of sound patterns that, when altered, characterize a central auditory processing disorder (CAPD)^(5)^. CAPD can occur as an isolated diagnosis or in combination with other diagnoses, so multidisciplinary monitoring is important in many cases^(6)^.

The diagnosis of CAPD often reveals difficulties in speech understanding, language development and learning^(6)^. Considering that CAP allows the maintenance of auditory performance when acoustic signals are degraded or with competing environmental sounds^(4)^, the disadvantages associated with CAPD become even more relevant when the individual is in challenging listening environments with a direct impact on social interaction and learning^(5)^.

After the diagnosis, our main action is to perform acoustically controlled auditory training (ACAT), which is based on the neural plasticity that is characteristic of the auditory system. Neural plasticity can be briefly described as the changes that occur in nerve cells due to environmental influences and physiological maturation to which all humans undergo, especially during the first years of life^(7)^.

However, if such influences can be controlled and adjusted to occur in a certain way, the auditory behavior related to plasticity can become predictable^(8)^. Then, ACAT can promote neurophysiological changes, with a positive impact on the auditory behavior^(9)^.

The literature recognizes the efficacy of auditory training programs^(5)^, as reported in some studies that investigated CAPD and ACAT associated with other clinical conditions such as recurrent otitis media^(10)^ and psychiatric diagnoses, such as Attention Deficit Hyperactivity Disorder (ADHD), and dyslexia^(11)^. These studies have demonstrated that, even in children with such clinical conditions, this intervention is valid and can have a significant impact on learning processes.

The use of software in ACAT has been a great ally to engage children in the therapeutic process^(12)^. In this strategy, altered auditory skills are stimulated through computer games, which significantly motivates children undergoing this type of intervention^(10)^.

The benefits of ACAT have been strongly described in the literature^(5)^, but almost always in a specific context of post-training evaluation and reevaluation (cross-sectional studies), without monitoring the evolution or maintenance of such skills using a longitudinal approach. Few studies have investigated the benefits from a longitudinal perspective to assess the maintenance of skills in the short, medium and long term^(8,11)^.

## METHODS

This is a randomized clinical trial. It was approved by the Research Ethics Committee of the institution, under approval no. 9205122.2.0000.5404. Data analysis was conducted using a quantitative descriptive analysis approach.

Data collection took place between October 2022 and January 2024 at a state school and at the university’s Audiology Laboratories. To select the sample, an invitation letter was attached to every child’s school agenda and sent to the guardians, requesting a written authorization to participate in the study. The students were evaluated only after their guardians signed an informed consent form and participants an assent form.

**Inclusion criteria**

Subjects were selected according to the following inclusion criteria:

Age group: 8 to 13 years old, native speakers of Brazilian Portuguese.
No history of speech therapy intervention and no diagnosis of neurodevelopmental disorders (information collected with the pedagogical team or at the time of history taking from guardians).
Absence of alterations in the external auditory canal, middle ear, recurrent otitis media and/or hearing loss confirmed or identified by audiological evaluation.
Impaired school performance (percentile less than or equal to 40) in at least one of the subtests (reading and/or writing) of the Teste de Desempenho Escolar II (TDE II)^(13)^.
Adequate score on the Teste de Matrizes Progressivas Coloridas de Raven^(14)^, indicating appropriate intellectual performance for the age group.
Diagnosis of central auditory processing disorder, with at least two altered test results in the test battery.
Compliance with the auditory training assessment and reassessment cycle proposed in the study methodology.
**Exclusion criteria**

The following exclusion criteria were considered in this study:

Speech disorders, craniofacial disorders and/or diagnoses of neurodevelopmental disorders.
History of speech therapy intervention focused on CAPD and/or oral and/or written language.
Presence of cognitive and neurological alterations, alterations of the external auditory canal and middle ear and/or confirmed hearing loss.
**Composition of study groups**

A total of 438 letters were sent, of which 248 were signed and sent back, allowing the students to participate in this study. In the initial assessment, 84 subjects fit the profile of impaired performance in at least one of the TDE II subtests. However, 64 subjects ended up being excluded due to the following reasons:

13 subjects presented intellectual performance below expectations in the Teste de Matrizes Progressivas Coloridas de Raven;
2 subjects had diagnoses of neurodevelopmental disorders;
12 subjects had peripheral auditory alterations and/or a history of recurrent otitis media,
1 subject had a submucous cleft palate;
7 subjects had phonological disorders;
16 subjects had a history of speech therapy intervention,
2 subjects did not show any alterations in the CAP assessment;
11 subjects had guardians who were not interested in participating in the study.
**Final sample**

The final sample consisted of 20 schoolchildren aged 8 to 13 years with impaired performance in the school screening, diagnosed with CAPD, and who fulfilled the other selection criteria. Subjects were randomly divided into two groups described below:

***Intervention group (IG):*** 10 subjects who underwent 8 sessions of acoustically controlled auditory training with subsequent reassessments immediately after the intervention and again 3 months after the end of the auditory training.
***Control group (CG):*** 10 subjects who did not undergo acoustically controlled auditory training and had a new CAP assessment 3 months after the first assessment. All subjects in the control group were invited to undergo auditory training after this monitoring process in case the altered skills remained.
**Selection procedures**

- School performance screening

To select subjects with impaired school performance, the Teste de Desempenho Escolar II (TDE II)^(13)^ were used. These tests were applied at the school, in a room specifically designated for the screening. In the writing subtest, the child was asked to write 40 words dictated by the researcher the way they judged to be correct, without support or tips. In the reading subtest, the child was asked to read a list of 36 words.

Based on the number of correct answers the subject received in these subtests, the protocol provided a performance classification according to what is expected for their school year. All subjects in this study fell into percentiles lower than or equal to 40 points in at least one of the reading and/or writing subtests of the TDE II, with their school performance characterized by the protocol as at least “medium-lower performance,” which in this study was considered “impaired performance.”

- Cognitive screening

After the reading and writing tests, subjects who fit the percentile established as inclusion criteria for the study underwent a cognitive screening. This test was also performed at the school, in a room specifically designated for the screening. Teste de Matrizes Progressivas Coloridas de Raven^(14)^ was applied and analyzed by a volunteer neuropsychologist, as another step performed before the CAP assessment. This test evaluates nonverbal intelligence skills using incomplete images that must be completed by the child by choosing the complement drawing from six options. The subject’s score was later transformed into a percentile that reflects the child’s eductive ability. Subjects who did not achieve the appropriate score for their age were excluded from the subsequent stages of the study.

- Interview and history taking

Subjects who met the inclusion criteria after the cognitive screening and the TDE II, were later invited to a meeting at the University Audiology Laboratories, where an interview was conducted and a history was taken from the subjects and guardians to collect data on the child’s history.

As part of data collection for sample characterization, the Scale of Auditory Behaviors (SAB) was applied^(15)^. This scale was selected due to its ability to ensure access to qualitative information that may be associated with CAPD involving everyday situations and its sensitivity to identify subjects at risk for diagnosis^(16)^. The protocol was applied twice – the first time with the responses about the perception of the child’s guardian, and the second time to collect the child’s self-perception of difficulties related to CAP in their daily life.

- Assessment of the peripheral auditory system

After the initial interview, a basic audiological evaluation was performed. Pure-tone audiometry and speech audiometry tests were performed in a soundproof booth with an AC40 audiometer and TDH39 headphones. Pure-tone audiometry involved the investigation of the thresholds at frequencies of 250 Hz, 500 Hz, 1 KHz, 2 KHz, 3 KHz, 4 KHz, 6 KHz, and 8 KHz. As a criterion of normality, the parameter of average thresholds obtained at the frequencies of 500 Hz, 1 KHz, 2 KHz, and 4 KHz ≤15 dB^(17)^ was adopted

For the speech audiometry tests, the speech recognition threshold (SRT) was investigated (considered as within the expected value equal to or up to 10 dBHL above the average (500 Hz, 1 KHz, and 2 KHz)^(18)^. To calculate the speech recognition percentage index (SRPI), a list of monosyllabic words at 40 dB above the tritonal average obtained in the audiometry was used. The responses obtained were considered as within normal limits when they achieved at least 92% accuracy in the identification of the words^(18)^.

To perform the immittance testing (tympanometry and acoustic reflexes), Interacoustics AT235 device was used to assess the functional integrity of the tympanic-ossicular system. Type A tympanometric curves were considered normal when indicating adequate mobility of the tympanic-ossicular system (volume: 0.30 to 1.65 ml; peak pressure: around 0 daPa, with a possible deviation of up to −100 daPa)^(19)^. Acoustic reflexes were assessed at frequencies of 500 Hz, 1 KHz, 2 KHz, and 4 KHz, ipsilaterally and contralaterally; normality was considered when ipsilateral reflexes were present^(20)^.

- Assessment of central auditory processing

All subjects in the sample (control and intervention groups) underwent a battery of tests for the diagnosis of CAPD, which was named *Timepoint A*.

For the assessment of central auditory processing, conducted in the University Audiology Laboratories, we considered the diagnosis of CAPD when there were at least two tests outside the normality criterion.

The following battery of tests was performed^(21)^:

Dichotic digit test (DDT)
Normality criterion: ≥95% accuracy bilaterally (9 years) and 85% (RE) and 82% (LE) (8 years).
Synthetic sentence identification (SSI) test with competitive message. Before applying this test, the reading and comprehension ability was assessed in relation to the demand offered.
Normality criterion (Signal/Noise Ratio - 15) ≥60%.
Dichotic consonant vowel (DCV) test
Normality criterion 8-12 years (free attention): minimum number of accuracy; RE: 8 and LE: 4; maximum number of errors: 6.
Frequency pattern test (FPT)^(22)^
Normality criterion: score ≥47% for 8-year-old children, 62% for children between 9 and 10 years old, 69% for children between 11 and 12 years old, and 75% for children between 13 and 14 years old.
Gaps in noise (GIN)^(23)^
Normality criterion: ≤6.1 ms bilaterally.

- Acoustically controlled auditory training

The acoustically controlled auditory training sessions took place at the University Audiology Laboratories, in meetings previously scheduled with the guardians. Eight weekly sessions of 45 minutes each were held, using activities from the *Afinando o Cérebro* platform for the intervention to stimulate central auditory processing skills.

The auditory training was performed in a soundproof booth, using headphones and the Interacoustics AC40 clinical audiometer connected to a laptop with access to the *Afinando o Cérebro* platform. A single protocol was adopted for all children and the sessions were planned and performed to stimulate figure-ground skills (monotonic and dichotic listening), temporal resolution, binaural integration, and temporal ordering.

The principles of the ACAT protocol adopted in this study aim to motivate the child to perform auditory tasks presented at levels of difficulty appropriate to their age group and individual characteristics. Therefore, the stimuli were presented at 50 dB above the tritonal average (500 Hz, 1000 Hz, and 2000 Hz) in order to ensure the child’s understanding of requested tasks. The difficulty levels progressed according to scores above 80% in order to encourage the interest in progressing through the auditory tasks^(24)^.

- Reassessment

The subjects in the IG were reassessed immediately after the 8 sessions of the auditory training (*Timepoint B*), while the subjects in the CG were reassessed 3 months after diagnosis, without any type of intervention (*Timepoint C*). Finally, three months after the end of the intervention (*Timepoint D*), the subjects in the IG were reassessed for the last time. In all evaluations, the same battery of tests presented in item D was performed.

**Statistical analysis**

The results were added to spreadsheets and submitted to descriptive statistical analysis (Statistical Analysis System 9.4) with the development of frequency tables for categorical variables and measures of position and dispersion for numerical variables. Fisher’s exact test was used to compare proportions, and the Mann-Whitney test was used to compare numerical measures between the two groups. The McNemar test was used to compare proportions evaluated at two timepoints. The Wilcoxon test for related samples was used to compare the test results between two timepoints. The Friedman test was used to compare the test results at three timepoints, followed by the Dunn test to find differences, when necessary. The level of significance adopted for the statistical tests was 5% and data that met this condition are highlighted in bold in the tables.

## RESULTS

The results were presented in items in the following order: sample characterization, Timepoint A of the CG and IG, comparison between Timepoints A and C of the CG, comparison of Timepoints A, B, and D of the IG, and finally, the qualitative comparative analysis between Timepoints A and C of the CG and Timepoints B and D of the IG.

Table 1 shows data of the composition of the CG and IG regarding age, sex, and school grade. Data obtained by the groups in the school performance screening (TDE test) and in the cognitive screening (Raven’s test) are also described, as well as the results obtained in the application of the SAB protocol. No statistically significant difference was observed between the groups, which highlighting the sample homogeneity.

**Table 1:** Sample characterization

**Parameter**		**Control group (N=10)**	**Intervention group (N=10)**	**Total (N=20)**	**p-value**
**Sex**	**Female**	5 (50.0%)	8 (80.0%)	13 (65.0%)	0.3498^2^
	**Male**	5 (50.0%)	2 (20.0%)	7 (35.0%)	-
**Age**		9.10 ± 0.88	9.40 ± 1.07	9.25 ± 0.97	0.5206^1^
**RAVEN’s Test**		29.20 ± 2.94	29.80 ± 2.25	29.50 ± 2.56	0.8476^1^
**School grade**	**3rd grade**	4 (40.0%)	0 (0.0%)	4 (20.0%)	-
	**4rd grade**	4 (40.0%)	5 (50.0%)	9 (45.0%)	-
	**5rd grade**	2 (20.0%)	4 (40.0%)	6 (30.0%)	-
	**7rd grade**	0 (0.0%)	1 (10.0%)	1 (5.0%)	-
**Reading Subtest (TDE II)**	**Accuracy in Reading**	30.60 ± 5.06	31.50 ± 4.33	31.05 ± 4.61	0.7899^1^
	**Percentile in Reading**	41.75 ± 32.92	30.30 ± 31.34	36.03 ± 31.83	0.2850^1^
**Writing Subtest (TDE II)**	**Accuracy in Writing**	20.30 ± 9.65	23.80 ± 8.02	22.05 ± 8.82	0.4952^1^
	**Percentile in Writing**	18.60 ± 15.26	30.00 ± 21.63	24.30 ± 19.14	0.1713^1^
**SAB Protocol**	**SAB score of guardian**	39.80 ± 8.80	37.90 ± 7.61	38.85 ± 8.07	0.7613^1^
	**SAB score of child**	38.50 ± 8.78	37.60 ± 7.92	38.05 ± 8.15	0.8794^1^

1 Based on the Mann-Whitney test;

2 Based on the Fisher’s test. Mean ± standard deviation

Table 2 shows data of each test of the battery applied to both control and intervention groups. A statistically significant difference was observed in the comparison of the performance of both groups in the SSI Test – signal/noise ratio (-15) in the right ear (RE).

**Table 2:** Results of CAP tests at Timepoint A for control and intervention groups

**Tests – 1^st^ Assessment of CAP – Timepoint A**	**Control Group (N=10)** (Mean ± Standard Desviation)	**Intervention Group (N=10)** (Mean ± Standard Desviation)	**Total (N=20)** (Mean ± Standard Desviation)	**p-value**
**DDT RE**	94.00 ± 5.03	93.88 ± 5.05	93.94 ± 4.90	0.9693^1^
**DTD LE**	95.50 ± 4.38	95.38 ± 2.89	95.44 ± 3.61	0.7579^1^
**DCVT – AL RE**	9.60 ± 2.41	9.00 ± 2.91	9.30 ± 2.62	0.4917^1^
**DCVT – AL LE**	8.90 ± 2.85	7.30 ± 1.77	8.10 ± 2.45	0.2036^1^
**SSI - S/R (-15) RE**	52.00 ± 20.98	35.00 ± 8.50	43.50 ± 17.85	**0.0290^1^**
**SSI - S/R (-15) LE**	45.00 ± 29.91	39.00 ± 13.70	42.00 ± 22.85	0.9693^1^
**FPT RE**	50.62 ± 30.49	64.65 ± 17.81	57.64 ± 25.35	0.2725^1^
**FPT LE**	48.33 ± 30.15	59.33 ± 26.36	53.83 ± 28.14	0.3250^1^
**GIN RE**	4.60 ± 1.07	4.00 ± 1.56	4.30 ± 1.34	0.3837^1^
**GIN LE**	4.50 ± 1.08	4.20 ± 1.48	4.35 ± 1.27	0.6781^1^

1 Basead on Mann-Whitney Test

**Caption:** DDT: Dichotic digit test, DCVT - FA: Dichotic consonant vowel test - Free attention, SSI –S/N ratio: Synthetic sentence identification test - Signal to noise ratio (-15), FPT: Frequency pattern test, GIN: Gaps-in-Noise Test, RE: Right ear, LE: Left ear

Table 3 shows data of the comparison between the diagnostic evaluation of CAP (Timepoint A) and the reassessment after 3 months without intervention (Timepoint C) of the CG. A statistically significant difference was observed in the percentage of accuracy in the following tests: DDT and DCV in the RE, FPT in the left ear (LE), and SSI and GIN in both ears.

**Table 3:** Comparative results of CAP tests at Timepoint A and C for the control group

**Tests**			**N**	**Mean**	**Standard deviation**	**Min.**	**Median**	**Max.**	**p-value** *
**DDT**	**RE**	Timepoint A	10	94.0%	5.0	85.0	93.8	100.0	
		Timepoint C	10	99.0%	2.4	92.4	100.0	100.0	
		Difference	10	5	4.1	0	6.2	10.0	**0.0156**
	**LE**	Timepoint A	10	95.5%	4.4	87.5	96.3	100.0	
		Timepoint C	10	88.8%	30.9	87.5	100	100.0	
		Difference	10	-7.4	32.6	-7.5	2.5	10.0	0.5547
**DCVT**	**RE**	Timepoint A	10	9.6	2.4	6.0	10.5	13.0	
		Timepoint C	10	12.3	1.6	10.0	12.0	15.0	
		Difference	10	3.0	2.7	-1	3.0	8.0	**0.0117**
	**LE**	Timepoint A	10	8.9	2.8	5.0	8.5	15.0	
		Timepoint C	10	7.4	2.8	1.0	8.0	11.0	
		Difference	10	-1.5	3.7	-9.0	-0.5	2.0	0.4766
**SSI- S/R (-15)**	**RE**	Timepoint A	10	52.0%	21.0	30.0	50.0	100.0	
		Timepoint C	10	67.0%	18.9	40.0	60.0	90.0	
		Difference	10	15.0%	17.2	-10.0	20.0	40.0	**0.0273**
	**LE**	Timepoint A	10	45.0%	29.9	10.0	40.0	90.0	
		Timepoint C	10	71.0%	20.8	40.0	65.0	100.0	
		Difference	10	26.0%	29.5	-30.0	25.0	80.0	**0.0313**
**FPT**	**RE**	Timepoint A	10	50.6%	30.5	13.3	43.1	100.0	
		Timepoint C	10	56.0%	30.4	23.3	41.6	100.0	
		Difference	10	5.4%	5.1	-13.3	3.3	33.4	0.3789
	**LE**	Timepoint A	10	48.3%	30.2	16.7	36.6	100.0	
		Timepoint C	10	58.0%	29.5	16.7	50.0	100.0	
		Difference	10	9.7%	10.8	-0.1	.4	33.3	**0.0313**
**GIN**	**RE**	Timepoint A	10	6.0 ms	2.4	2.0	5.0	10.0	
		Timepoint C	10	4.6 ms	1.1	2.0	5.0	6.0	
		Difference	10	-1.4 ms	1.8	-10.0	-0.5	0.0	**0.0625**
	**LE**	Timepoint A	10	5.9 ms	3.5	2.0	5.0	15.0	
		Timepoint C	10	4.5 ms	1.1	2.0	5.0	5.0	
		Difference	10	-1.4 ms	3.1	-10.0	-0.5	0.0	**0.0625**

* Based on the Wilcoxon tests for related samples

**Caption:** DDT: Dichotic digit test, DCVT - FA: Dichotic consonant vowel test - Free attention, SSI –S/N ratio: Synthetic sentence identification test - Signal to noise ratio (-15), FPT: Frequency pattern test, GIN: Gaps-in-Noise Test, RE: Right ear, LE: Left ear

Table 4 shows the results of the comparison of Timepoints A, B, and D (IG). A statistically significant difference was observed in the following tests: DDT in the RE and LE – Timepoints AxD; DCV in the RE – Timepoints AxB and AxD, SSI in the RE and LE – Timepoints AxB and AxD; FPT in the RE – Timepoints AxB and BxD; FPT in the LE – Timepoints AxB; GIN – Timepoints AxB and AxD in both ears.

**Table 4:** Results of CAP tests for diagnosis (Timepoint A), post-ACT reassessment (Timepoint B), and three months after the auditory training (Timepoint D) for the IG

**Tests**			**N**	**Mean**	**Standard deviation**	**Min.**	**Median**	**Max.**	**p-value** 2	p-value* **(AxB)**	p-value* (AxD)	p-value* (BxD)
**DDT**	**RE**	Timepoint A	10	93.9%	5.1	83.8	93.8	100.0				
		Timepoint B	10	99.0%	1.7	95.0	100.0	100.0				
		Difference	10	5.1	5.4	-1.3	100.0	16.3	**0.0156** 1			
		Timepoint D**	10	100%	0.00	100.0						
	**LE**	Timepoint A	10	95.4%	2.9	92.5	95.0	100.0				
		Timepoint B	10	97.8%	2.2	95.0	97.5	100.0				
		Timepoint D	10	99.5%	1.6	95.0	100.0	100.0	**0.0005^2^**	**0.0451**	**<0.0001**	0.0842
**DCVT**	**RE**	Timepoint A	10	9.00	2.9	5.0	9.5	14.0				
		Timepoint B	10	11.0	1.9	8.0	11.5	13.0				
		Timepoint D	10	12.4	2.6	8.0	12.0	17.0	**0.0482**	0.3816	**0.0469**	0.0639
	**LE**	Timepoint A	10	7.3	1.8	4.0	8.0	10.0				
		Timepoint B	10	8.0	2.8	5.0	8.0	12.0				
		Timepoint D	10	7.6	2.1	5.0	7.5	12.0	0.2354	---	---	---
**SSI S/R (-15)**	**RE**	Timepoint A	10	35.0%	8.5	20.0	35.0	50.0				
		Timepoint B	10	71.0%	13.7	60.0	70.0	100.0				
		Timepoint D	10	74.0%	10.7	60.0	70.0	100.0	**<0.0001**	**<0.0001**	**<0.0001**	0.2789
	**LE**	Timepoint A	10	39.0%	13.7	20.0	40.0	60.0				
		Timepoint B	10	66.0%	9.7	60.0	60.0	90.0				
		Timepoint D	10	69.0%	12.9	60.0	65.0	100.0	**<0.0001**	**<0.0001**	**<0.0001**	0.8638
**FPT**	**RE**	Timepoint A	10	64.7%	17.8	30.0	66.7	86.6				
		Timepoint B	10	81.4%	13.8	60.0	83.3	100.0				
		Timepoint D	10	76.3%	14.8	56.6	71.7	96.7	**0.0105**	**0.0044**	0.3371	**0.0220**
	**LE**	Timepoint A	10	59.3%	26.4	16.7	56.6	100.0				
		Timepoint B	10	78.7%	11.8	63.3	76.9	100.0				
		Timepoint D	10	77.7%	12.1	63.3	73.5	93.3	**0.0345**	**0.0393**	0.1027	0.2419
**GIN**	**RE**	Timepoint A	10	5.7 ms	2.2	2.0	6.0	10.0				
		Timepoint B	10	4.0 ms	1.6	2.0	4.0	6.0				
		Timepoint D	10	4.0 ms	1.4	2.0	4.5	6.0	**0.0343**	**0.0522**	**0.0067**	1.0000
	**LE**	Timepoint A	10	6.5 ms	3.3	2.0	5.5	12.0				
		Timepoint B	10	4.2 ms	1.5	2.0	4.5	6.0				
		Timepoint D	10	4.2 ms	1.5	2.0	5.0	6.0	**0.1003**	**-----------**	**-----------**	**-----------**

1 Based on the Wilcoxon tests for related samples;

2 Based on the Friedman test, followed by the Dunn test (*) to locate the differences;

** No variability at this time, not included in the comparative analysis

**Caption:** DDT: Dichotic digit test, DCVT - FA: Dichotic consonant vowel test - Free attention, SSI –S/N ratio: Synthetic sentence identification test - Signal to noise ratio (-15), FPT: Frequency pattern test, GIN: Gaps-in-Noise Test, RE: Right ear, LE: Left ear

Table 5 shows the categorical qualitative results (normal versus altered), considering the assessment and reassessment of both groups with a 3-month interval. Of all 10 subjects in the CG, 6 maintained altered tests after the 3-month interval between assessments. In the IG, no difference was observed between both evaluations, with maintenance of the performance observed immediately after the ACAT and 3 months after the end of the intervention.

**Table 5:** Qualitative results of the CAP assessments, for the control and intervention groups

**CAP test**		**Control group Comparison: Timepoints A x C**				**Intervention group Comparison: Timepoints B x D**			
		**Frequency(%)**	**Altered**	**Normal**	**Total**	**Frequency(%)**	**Altered**	**Normal**	**Total**
**DDT**	**RE**	**Altered**	1 - 10.%	4 - 40%	5 - 50%	**Altered**	0 - 0%	0 - 0%	0 - 0%
		**Normal**	0 - 0%	5- 50%	5 - 50%	**Normal**	0 - 0%	10 -100%	10-100%
	**LE**	**Altered**	1- 10%	2- 20%	3 - 30%	**Altered**	0 - 0%	0 - 0%	0 - 0%
		**Normal**	1-10%	6- 60%	7 - 70%	**Normal**	0 - 0%	10 -100%	10-100%
**DCVT**	**RE**	**Altered**	0-0%	1 - 10%	1 - 10%	**Altered**	0 - 0%	0 - 0%	0 - 0%
		**Normal**	0-0%	9 - 90%	9 - 90%	**Normal**	0 - 0%	10 -100%	10-100%
	**LE**	**Altered**	0-0%	0 - 0%	0 - 0%	**Altered**	0 - 0%	0 - 0%	0 - 0%
		**Normal**	0-0%	10 - 100%	10-100%	**Normal**	0 - 0%	10 -100%	10-100%
**SSI S/R (-15)**	**RE**	**Altered**	3- 30%	4-40%	7- 70%	**Altered**	10-100%	10-100%	10-100%
		**Normal**	0 - 0%	3 – 30%	3 - 30%	**Normal**	0 - 0%	10-100%	10-100%
	**LE**	**Altered**	2 - 20%	5 - 50%	7 - 70%	**Altered**	0 - 0%	0 - 0%	0 - 0%
		**Normal**	0 - 0%	3- 30%	3 - 30%	**Normal**	0 - 0%	10-100%	10-100%
**FPT**	**RE**	**Altered**	6 - 60%	0 - 0%	6 - 60%	**Altered**	0 - 0%	0 - 0%	0 - 0%
		**Normal**	0 - 0%	0 - 40%	4 - 40%	**Normal**	0 - 0%	10-100%	10 - 100%
	**LE**	**Altered**	6 - 60%	0 - 0%	6 - 60%	**Altered**	0 - 0%	0 - 0%	0 - 0%
		**Normal**	0 - 0%	0 - 40%	4 - 40%	**Normal**	0 - 0%	10-100%	10-100%
**GIN**	**RE**	**Altered**	0 - 0%	6- 60%	6- 60%	**Altered**	0 - 0%	0 - 0%	0 - 0%
		**Normal**	0 - 0%	4 - 40%	4 - 40%	**Normal**	0- 0%	10-100%	10-100%
	**LE**	**Altered**	0 - 0%	4 - 40%	4 - 40%	**Altered**	0 - 0%	0 - 0%	0 - 0%
		**Normal**	0 - 0%	6 - 60%	6 - 60%	**Normal**	0 - 0%	10-100%	10-100%

**Caption:** DDT: Dichotic digit test, DCVT - FA: Dichotic consonant vowel test - Free attention, SSI –S/N ratio: Synthetic sentence identification test - Signal to noise ratio (-15), FPT: Frequency pattern test, GIN: Gaps-in-Noise Test, RE: Right ear, LE: Left ear

## DISCUSSION

There is an important debate in the literature and in clinical practice about the extent to which we can relate the evolution of auditory skills to neurophysiological maturation or to the improvements obtained after ACAT at different assessment timepoints with the same subject? This reality motivated our study and supports our interest in understanding the differences between each assessment timepoints with each group.

Regarding the distribution of the sample in terms of sex, age, school grade, and school performance, we observed that both CG and IG were considered homogeneous (Table 1). This finding is relevant because it shows no influence of variables of sex, age, school grade, and school performance on the results. The same things was observed for data from the first assessment (Timepoint A) of both groups (Table 2), except in the SSI test, in the signal/noise ratio (-15), in which the CG had a better performance when compared to the IG.

When analyzing data of the comparison between Timepoint A and Timepoint C of the CG (Table 3), a statistically significant difference was observed between these timepoints. The improvement in the accuracy percentage reached 26%, observed in the SSI test of the LE. Another important aspect was that not all tests showed an improvement in the accuracy percentage – a negative difference was observed in the comparison of Timepoint A and Timepoint C in the DDT and DCV tests of the LE. These subjects did not undergo speech therapy intervention in the interval between both assessments. This difference can also be justified by the neuromaturation of auditory skills that will reach maturity only between 12 and 13 years of age^(25)^.

Concomitantly, the better performance of the CG at Timepoint C can also be related to the stimulation of auditory skills associated with the sound experiences of these subjects in the interval between the first and second assessments^(1)^. This scenario was also found in a study with children in the early stages of literacy who underwent CAP assessment and reassessment with a 6-month interval and without speech therapy intervention. A statistically significant improvement was observed in tests involving auditory skills of sound localization, figure-ground skills for linguistic sounds and temporal resolution^(26)^. However, it is important to highlight that, in our study sample, this stimulation of auditory skills, part of everyday life in the auditory world, was not enough for all subjects to reach normality according to their age groups. Of all 10 subjects of the CG, 60% of them did not reach the normality criteria at Timepoint C.

Now regarding the comparative analysis of the results of the IG at Timepoints A, B, and D (Table 4), an improvement was observed in CAP skills after ACAT and maintenance of such improvement in the reassessment 3 months after the end of the intervention. A statistically significant difference was observed in the DDT, DCV (RE only), SSI, FPT, and GIN tests. In addition, contrary to the results observed in the comparative analysis of Timepoints A and C of the CG, the mean values obtained at each assessment timepoint gradually increased (except for the GIN test, in which the decrease in the mean value reflects an improvement in the temporal resolution ability).

Considering the values observed at Timepoints A and B for the IG, pre- and post-ACAT, we observed a significant increase in the mean values due to an improvement in the auditory skills after ACAT. An increase in mean scores was also observed in the DDT, DCV (for the RE), SSI, and FPT tests. When comparing the mean values obtained at Timepoints B and D 3 months after the end of ACAT, these values remained the same or even increased (except for the FPT, but it remained within the normality criteria). These findings support our hypothesis of the effectiveness of ACAT and the maintenance of the improved skills with a longitudinal assessment approach.

Other studies have also recognized the effectiveness of auditory training in children with impaired school performance^(27)^, phonological disorders, and even a history of otitis media. Some studies analyze the effectiveness of ACAT in children with impaired school performance diagnosed with dyslexia^(28)^ and attention deficit hyperactivity disorder (ADHD), with ACAT effectively adapting altered auditory skills. ACAT was also efficient in children with a history of phonological disorders, with evidence of improvement and adaptation of auditory gnosis processes after ACAT^(29)^. In investigations assessing the effectiveness of ACAT in schoolchildren with recurrent otitis media^(10)^, the results highlighted that ACAT triggered a statistically significant difference for all behavioral tests in the pre- and post-ACAT comparison, with better performance after the intervention in temporal ordering, temporal resolution and figure-ground tests.

The effectiveness of ACAT is also recognized in children with CAPD but without impaired school performance who underwent intervention^(9)^. One study evaluated 30 children whose CAP assessment results included at least two altered tests. After ACAT, only 11 children had scores below expected and 19 children achieved scores according to the normality criteria^(8)^.

Maintenance of skills acquired after ACAT intervention has been analyzed in other studies which reported the maintenance of auditory skills using a longitudinal approach. Schochat et al.^(30)^, using a longitudinal design, analyzed 20 individuals aged 8 to 24 years who underwent auditory training. In the reassessment, 85% of these subjects improved with ACAT and maintained such improvements over time, supporting the effectiveness of ACAT in most of the cases analyzed. Another study assessed 10 individuals aged 7 to 14 years in the first assessment, and 8 to 17 years in the last reassessment, observed an improvement in skills after ACAT and the maintenance of such results three years after the end of the stimulation^(31)^.

The CAP assessment tests are considered in the literature as stable tests, as demonstrated in test-retest studies in individuals with typical development^(32)^. Therefore, changes in responses at different timepoints of assessment may reflect changes in the auditory system and its functions. Such changes may be the result of factors such as compensation after injury, maturation of the auditory system in early childhood, or even learning facilitated by a particular intervention.

In our study, considering the short interval between the assessment timepoints (8 weeks between Timepoints A and B and 3 months between Timepoints B and D) and the maintenance of these results even after the end of the intervention in the IG, we concluded that the changes observed in the performance of subjects between each assessment are associated with learning facilitated by ACAT. Our conclusion is based on the fact that the 8-week interval would be insufficient to relate such changes only to the maturation of the auditory system, as observed in the study by Filippini et al.^(31)^.

As indicated in Table 5, all subjects in the IG maintained the results within the expected range after the end of the speech-language intervention through the ACAT; while in the CG, the 3-month interval was not sufficient for all subjects with altered auditory skills to reach the normal standard that is appropriate for their age group. Six subjects maintained scores below the expected range and were invited to perform the ACAT after the reassessment. These data support the clinical recommendation of ACAT, since the physiological maturation of auditory skills may have its normal course modified and may even not reach full maturity. Given the impact of changes in auditory skills on the child’s language development, ACAT aims to adapt the analysis and interpretation of auditory stimuli, promoting the process of maturation of the central auditory nervous system to help improve school performance.

Given the influence of auditory skills on school performance and the academic learning process, the child should have access to stimulation of auditory skills at an appropriate time so that they can achieve full performance during this process, minimizing reading and writing difficulties and delays in the school training.

We highlight that future studies should investigate the effectiveness and maintenance of auditory skills acquired after ACAT for longer periods of post-intervention monitoring. In addition, these studies should include a larger sample size. Future studies should focus on comparing CAP reassessment data with not only behavioral tests, but also electrophysiological tests to investigate the effects of ACAT.

## CONCLUSION

Based on the analysis of the results, we concluded that ACAT effectively improved the auditory skills of children with impaired school performance and maintained the skills acquired in a reassessment conducted 3 months after the end of the intervention.
