# Supplementary material for: Short-term effects of acoustically controlled auditory training in children with impaired school performance
Source: Codas. 2025 May 26;37(3):e20240135. doi: 10.1590/2317-1782/e20240135en (PMC12121903; doi:10.1590/2317-1782/e20240135en)
Supplement: Translation

# Translation: Efeitos à curto prazo do treinamento auditivo acusticamente controlado em crianças com desempenho escolar prejudicado

**DOI:** 10.1590/2317-1782/e20240135pt

**Authors:** Júlia Roja Tavoni, Maria Francisca Colella dos Santos

**Affiliations:** 1 Programa de Pós-graduação em Saúde da Criança e do Adolescente, Centro de Investigação em Pediatria, Universidade Estadual de Campinas – UNICAMP - Campinas (SP), Brasil.

**Keywords:** Transtornos da Percepção Auditiva, Plasticidade Neuronal, Reabilitação, Criança, Fonoaudiologia

## Abstract

**Objetivo:**

Investigar a efetividade do Treinamento Acusticamente Controlado (TAAC) e a manutenção das habilidades auditivas adquiridas por crianças com desempenho escolar prejudicado.

**Método:**

Amostra constituída por 20 escolares: Grupo Controle (GC – N=10) e Grupo Intervenção (GI – N=10), com idade entre 8 e 13 anos, resultados abaixo do esperado em uma triagem de desempenho escolar, resultados adequados em um rastreio cognitivo e diagnosticados com o Transtorno do Processamento Auditivo Central (TPAC) após bateria de testes comportamentais. O GC não recebeu nenhum tipo de intervenção, apenas avaliação e reavaliação após 3 meses. O GI foi avaliado imediatamente após 8 sessões de TAAC e após 3 meses da intervenção. Dados de avaliação de PAC foram submetidos à análise estatística (p<0,05).

**Resultados:**

A amostra foi considerada homogênea quanto ao sexo, idade, escolaridade, desempenho escolar. Quanto aos dados de avaliação de PAC, ambos os grupos eram homogêneos (com exceção do teste Identificação de Sentenças Sintéticas - SSI). As crianças do GI demonstraram melhora no desempenho dos testes Dicótico de Dígitos, Consoante Vogal, SSI e Padrão de Frequência (TPF) imediatamente após a intervenção. Após 3 meses do TAAC, tais valores se mantiveram ou aumentaram, exceto TPF. Na análise qualitativa, entre normal ou alterado, o GI manteve resultados adequados após 3 meses do TAAC. No GC, seis crianças mantiveram resultados alterados e quatro atingiram a normalidade na reavaliação pós 3 meses.

**Conclusão:**

O TAAC mostrou-se eficaz para a reabilitação das habilidades auditivas de crianças com desempenho escolar prejudicado mesmo após 3 meses do fim da intervenção.

## INTRODUÇÃO

O processo de aprendizagem tem como principal alicerce a linguagem, afinal, são as habilidades de linguagem expressiva e compreensiva que embasam o início da vida escolar. A integridade do sistema auditivo e das habilidades auditivas também é peça fundamental deste processo, pois é a partir dela que a criança tem acesso às informações do mundo que a rodeia, mesmo antes da aquisição da linguagem oral.

O desenvolvimento das habilidades auditivas se inicia logo na vida intrauterina, sendo estas adquiridas e aprimoradas ao longo do crescimento, a partir das experiências sonoras e da neuromaturação das estruturas do sistema nervoso central. As habilidades auditivas permitem que o indivíduo compreenda quais os sons relevantes em locais ruidosos, sustente a atenção auditiva por determinado período de tempo, compare, recupere e integre diferentes estímulos sonoros^(1)^.

Considerando o impacto da audição na aquisição da linguagem e a neuromaturação característica dos primeiros anos de vida, a maturação e o aprimoramento das habilidades auditivas impactam diretamente no desenvolvimento social, emocional e cognitivo da criança^(2)^. Ao mesmo tempo, crianças com alterações auditivas na primeira infância possuem maiores chances de enfrentar dificuldades na alfabetização e, consequentemente, no desenvolvimento das habilidades de leitura e escrita^(3)^.

Os processos e mecanismos do sistema nervoso auditivo foram definidos como pertencentes ao *Processamento Auditivo Central (PAC)*. Ao longo do trajeto do som pelas vias auditivas é que o indivíduo é capaz de detectar, localizar, reconhecer e, finalmente, interpretar a informação auditiva recebida^(4)^. Assim, o PAC é descrito como um conjunto de habilidades necessárias para a análise e interpretação de padrões sonoros que, quando encontram-se alteradas, caracterizam o Transtorno do Processamento Auditivo Central (TPAC)^(5)^. Destaca-se que o TPAC pode ocorrer como um diagnóstico isolado ou em comorbidade com outros diagnósticos, sendo importante o acompanhamento multiprofissional em muitos casos^(6)^.

O diagnóstico de TPAC frequentemente vem acompanhado de dificuldades de compreensão da fala, dificuldades no desenvolvimento da linguagem e, também, de aprendizagem^(6)^. Considerando-se que o PAC possibilita a manutenção do desempenho auditivo quando os sinais acústicos se encontram degradados ou com ruídos ambientais competitivos^(4)^, as desvantagens associadas ao TPAC tornam-se ainda mais relevantes quando o indivíduo se encontra em ambientes de escuta desafiadora com impacto direto na interação social e no aprendizado^(5)^.

Diante do diagnóstico, temos como principal repertório de ação a realização do Treinamento Auditivo Acusticamente Controlado (TAAC), baseado na plasticidade neural característica do sistema auditivo. A plasticidade neural pode ser resumida à mudança que ocorre nas células nervosas, reflexo de influências ambientais e da maturação fisiológica a qual todos os seres humanos estão sujeitos, principalmente nos primeiros anos de vida^(7)^.

Há porém de se considerar que, se tais influências podem ser controladas e ajustadas para ocorrerem de uma determinada forma, o comportamento auditivo relacionado à plasticidade pode se tornar previsível^(8)^. Assim o TAAC, é uma ferramenta capaz de favorecer mudanças neurofisiológicas impactando, positivamente, no comportamento auditivo^(9)^.

A literatura já reconhece que os programas de treinamento auditivo têm eficácia comprovada^(5)^. Há na literatura pesquisas que investigaram o TPAC e o TAAC associado a outros quadros clínicos como as otites de repetição^(10)^ e os diagnósticos psiquiátricos como o Transtorno de Déficit de Atenção e Hiperatividade (TDAH) e a Dislexia^(11)^. Comprovou-se que, mesmo em crianças com tais quadros clínicos, esta estratégia de intervenção é válida e pode ter impacto relevante nos processos de aprendizagem.

O uso de softwares para a realização do TAAC tem sido grande aliado para o engajamento das crianças no processo terapêutico^(12)^. Nessa estratégia as habilidades auditivas alteradas são estimuladas por meio de jogos computadorizados, fator de grande motivação para às crianças submetidas a este tipo de intervenção^(10)^.

Os benefícios desta intervenção são fortemente descritos na literatura^(5)^, contudo quase sempre em um contexto pontual de avaliação e reavaliação pós treinamento (estudos transversais), sem o monitoramento da evolução ou manutenção de tais habilidades em caráter longitudinal. Poucos estudos se dedicaram à investigação dos ganhos adquiridos numa perspectiva longitudinal para averiguação da manutenção das habilidades a curto, médio e longo prazo^(8,11)^.

## MÉTODO

Esta pesquisa se caracteriza como um estudo clínico randomizado. Foi submetida e aprovada pelo Comitê de Ética em Pesquisa da instituição, sob o protocolo nº 9205122.2.0000.5404. A análise dos dados obtidos foi realizada por meio de uma abordagem quantitativa, de cunho descritivo analítico.

A coleta de dados ocorreu entre Outubro de 2022 e Janeiro de 2024 em uma escola estadual e nos Laboratórios de Audiologia da universidade. Para seleção da amostra, foi anexada na agenda das crianças uma carta-convite direcionada aos responsáveis, solicitando a autorização por escrito para participação na pesquisa. Posteriormente, a avaliação dos alunos ocorreu apenas mediante assinatura também do Termo de Consentimento Livre e Esclarecido (TCLE) e Termo de Assentimento Livre e Esclarecido (TALE).

**Critérios de inclusão**

Os sujeitos foram selecionados de acordo com os **critérios de inclusão** descritos a seguir.

Faixa etária entre 8 e 13 anos e ter como língua materna o Português-Brasileiro.
Ausência de histórico intervenção fonoaudiológica prévia e ausência do diagnóstico de transtornos do neurodesenvolvimento (informações coletadas com a equipe pedagógica ou no momento de anamnese com responsáveis).
Ausência de alterações de meato acústico externo, orelha média, otites de repetição e/ou perdas auditivas confirmadas ou identificadas pela avaliação audiológica;
Desempenho escolar prejudicado (Percentil menor ou igual a 40) em pelo menos um dos subtestes (leitura e/ou escrita) do Teste de Desempenho Escolar (TDE II)^(13)^.
Pontuação adequada no Teste de Matrizes Progressivas de Raven^(14)^ indicando Desempenho Intelectual adequado à faixa etária.
Diagnóstico de Transtorno do Processamento Auditivo Central após a realização da bateria de avaliação com pelo menos 2 testes alterados.
Cumprimento do ciclo de avaliação treinamento auditivo e reavaliação proposto na metodologia da pesquisa
**Critérios de exclusão**

Já como **critérios de exclusão** foram considerados os seguintes aspectos:

Alterações de fala, alterações craniofaciais e/ou diagnósticos de Transtornos do Neurodesenvolvimento;
Sujeitos com histórico de intervenção fonoaudiológica prévia voltada para o TPAC e/ou Linguagem Oral e/ou Escrita;
Presença de alterações cognitivas e neurológicas, alterações de meato acústico externo, orelha média e/ou perdas auditivas confirmadas
**Composição dos grupos**

Ao todo foram enviadas 438 cartas, das quais 248 retornaram assinadas permitindo a participação dos escolares. Nesta avaliação inicial, 84 sujeitos encaixaram-se no perfil de desempenho prejudicado em pelo menos um dos subtestes do TDE II. Entretanto, 64 sujeitos acabaram excluídos devidos às seguintes justificativas:

13 sujeitos apresentaram desempenho intelectual abaixo do esperado no Teste de Matrizes Progressivas de Raven;
2 sujeitos apresentavam diagnósticos de transtornos do neurodesenvolvimento;
12 sujeitos apresentaram alterações auditivas periféricas e/ou histórico de otites de repetição,
1 sujeito apresentava fenda palatina submucosa;
7 sujeitos apresentavam desvios fonológicos;
16 sujeitos apresentaram histórico prévio de intervenção fonoaudiológica,
2 sujeitos não apresentaram alteração na avaliação de PAC;
11 sujeitos cujas famílias não demonstraram interesse em dar continuidade à participação na pesquisa.
**Amostra final**

A amostra final foi constituída por 20 escolares, entre 8 e 13 anos com desempenho prejudicado na triagem escolar, diagnosticados com o TPAC e demais critérios de seleção. Os sujeitos foram divididos aleatoriamente em dois grupos, descritos abaixo:

***Grupo Intervenção (GI):*** 10 sujeitos que passaram por 8 sessões do treinamento auditivo acusticamente controlado com subsequentes reavaliações logo após a intervenção e, novamente, após 3 meses da finalização do treinamento auditivo.
***Grupo Controle (GC):*** 10 sujeitos que não passaram pelo treinamento auditivo acusticamente controlado e repetiram a avaliação do PAC após 3 meses da primeira avaliação. Todos os sujeitos do grupo controle foram convidados a realizar o treinamento auditivo após este processo de monitoramento no caso de manutenção das habilidades alteradas.
**Procedimentos realizados**

- Rastreio do Desempenho Escolar

Para a seleção dos sujeitos com desempenho escolar prejudicado, foram adotados os Subtestes de Leitura e Escrita do Teste de Desempenho Escolar II^(13)^. Tais testes foram realizados nas dependências da escola, em uma sala designada para a realização da triagem. No subteste de escrita, a criança deveria escrever 40 palavras ditadas pela pesquisadora avaliadora da forma que julgava correto, sem apoio ou dicas. No subteste de leitura, a criança foi convidada a ler uma lista de 36 palavras.

Partindo do número de acertos da sujeito em tais subtestes, o protocolo traz uma classificação do desempenho com base no esperado para o seu ano escolar. Todos os sujeitos dessa pesquisa encaixaram-se em percentis menores ou iguais à 40 pontos em pelo menos um dos subtestes aplicados (leitura e/ou escrita) do Teste de Desempenho Escolar (TDE), tendo seu desempenho escolar caracterizado pelo protocolo como pelo menos “*desempenho médio- inferior”* que, neste estudo, denominamos como “desempenho prejudicado”

- Rastreio Cognitivo

Posteriormente à triagem de leitura e escrita, as sujeitos que se encaixaram no percentil estabelecido como critério de inclusão na pesquisa passaram por um rastreio cognitivo. Este teste também foi realizado nas dependências da escola, em uma sala designada para a realização da triagem. O Teste das Matrizes Progressivas Coloridas de Raven^(14)^ foi aplicado e analisado por uma neuropsicóloga parceira voluntária, como mais uma das etapas prévias à avaliação do Processamento Auditivo Central. Tal triagem se propõe a avaliar habilidades de inteligência não verbal por meio de imagens incompletas as quais a criança deve identificar entre seis opções qual o desenho que a completa. A pontuação do sujeito foi posteriormente transformada em um percentil que reflete a capacidade edutiva da criança. Os sujeitos que não atingiram a pontuação adequada para a idade foram excluídos das etapas seguintes do estudo.

- Entrevista e anamnese com os responsáveis

Os sujeitos que se encaixaram nos critérios de inclusão, considerando o rastreio cognitivo e o TDE II, foram convidados posteriormente a comparecer aos Laboratórios de Audiologia da Universidade. Neste encontro era realizada uma entrevista e anamnese com o sujeito e seus responsáveis para coleta de dados em relação a história pregressa da criança.

Como parte da coleta de dados focada na caracterização da amostra estudada, foi aplicada a Escala de Funcionamento Auditivo (Scale of Auditory Behavior – SAB)^(15)^. A escolha do uso deste questionário comportamental se baseia na sua capacidade de garantir o acesso à informações qualitativas que podem ter associação com o TPAC envolvendo situações cotidianas e a sua sensibilidade para identificar sujeitos com sinais de risco para o diagnóstico^(16)^. O protocolo foi aplicado duas vezes, sendo uma delas com as respostas referindo-se à percepção do responsável da criança e uma segunda vez com a própria criança, para averiguação da autopercepção da criança sobre as possíveis dificuldades relacionadas ao PAC em seu cotidiano.

- Avaliação do Sistema Auditivo Periférico

Após a entrevista inicial, era realizada a avaliação audiológica básica. Os testes audiometria tonal e a logoaudiometria foram realizados em cabina acústica com audiômetro AC40 e fones TDH39. A audiometria tonal envolveu a pesquisa dos limiares das frequências de 250Hz, 500Hz, 1KHz, 2KHz, 3KHz, 4KHz, 6KHz e 8KHz. Como critério de normalidade adotou-se o parâmetro de média dos limiares obtidos nas frequências de 500Hz, 1KHz, 2KHz e 4KHz ≤ 15 dB^(17)^.

Para logoaudiometria foram pesquisados o Limiar de Reconhecimento de Fala (LRF) (considerado como dentro do esperado valor igual ou até 10 dBNA acima da média (500Hz, 1KHz e 2KHz))^(18)^. Para realização do Índice Percentual de Reconhecimento de Fala (IPRF), foi utilizada uma lista de palavras monossilábicas apresentadas a 40 dB acima da média tritonal obtida na audiometria. As respostas obtidas foram consideradas como dentro da normalidade quando atingiam no mínimo 92% de acerto na identificação dos vocábulos apresentados^(18).^

Para a realização da imitanciometria (timpanometria e reflexos acústicos) foi utilizado o equipamento AT235 da Interacoustics visando-se a avaliação da integridade funcional do sistema tímpano-ossicular. Foram consideradas dentro da normalidade as curvas timpanométricas tipo A, que indicam a mobilidade adequada do sistema tímpano-ossicular (Volume: 0,30 a 1,65 ml Pressão pico: em torno de 0 daPa podendo desviar até -100daPa)^(19)^. Os reflexos acústicos foram pesquisados nas frequências de 500Hz, 1 KHz, 2 KHz e 4 KHz, ipsi e contralateralmente; tendo sido considerada a normalidade quando os reflexos ipsilaterais estivessem presentes^(20)^.

- Avaliação do Processamento Auditivo Central

Todos os sujeitos da amostra (Grupo Controle e Intervenção) foram submetidos à bateria de testes para o diagnóstico de TPAC que denominamos como *Momento A*.

Para Avaliação do Processamento Auditivo Central, realizada nos Laboratórios de Audiologia da Universidade, consideramos o diagnóstico de TPAC nos casos em que houve pelo menos dois testes fora do critério de normalidade.

Foi utilizada a seguinte bateria de testes^(21)^:

Teste Dicótico de Dígitos (TDD)
Critério de normalidade: ≥ 95% de acertos bilateralmente (9 anos) e 85% (OD) e 82% (OE) (8 anos).
Teste de Identificação de Sentenças Sintéticas com Mensagem Competitiva (SSI). Antes da aplicação deste teste foi checada a habilidade de leitura e compreensão quanto à demanda oferecida.
Critério de normalidade (Relação Sinal/Ruído -15) ≥60%.
Teste Dicótico Consoante Vogal (TDCV),
Critério de normalidade 8-12 anos (Atenção livre): número mínimo de acertos OD: 8 e OE: 4; número máximo de erros: 6
Teste de Padrão de Frequência (TPF)^(22)^
Critério de normalidade: pontuação ≥47% para crianças de 8 anos, 62% para crianças entre 9 e 10 anos de idade, 69% para crianças entre 11 e 12 anos de idade e 75% para idades entre 13 e 14 anos.
Gaps in Noise (GIN)^(23)^
Critério de normalidade: ≤ 6,1 ms bilateralmente.

- Treinamento Auditivo Acusticamente Controlado

As sessões de treinamento auditivo acusticamente controlado ocorreram nos Laboratórios de Audiologia da Universidade, em encontros previamente agendados com os responsáveis dos sujeitos. Foram realizados 8 encontros semanais de 45 minutos, com a utilização de atividades da Plataforma *Afinando o Cérebro* para intervenção quanto à estimulação das habilidades do processamento auditivo central.

O treinamento auditivo foi realizado em cabina acústica, com uso de fones de ouvido e o audiômetro clínico AC40 (Interacoustics) acoplado ao notebook com acesso à Plataforma Afinando o Cérebro. Foi adotado um protocolo único para todas as crianças e as sessões foram planejadas e realizadas com foco na estimulação das habilidades de figura-fundo (escuta monótica e dicótica), resolução temporal, integração binaural e ordenação temporal.

Os princípios adotados no protocolo de TAAC dessa pesquisa visam a motivação da criança durante as tarefas auditivas apresentadas em níveis de dificuldades adequados a sua faixa etária e as suas características individuais. Diante disso, os estímulos foram apresentados a 50dB acima da média tritonal (500Hz, 1000Hz e 2000Hz) como forma de garantir a compreensão da criança acerca das tarefas solicitadas. Os níveis de dificuldade foram progredidos a partir de pontuações superiores a 80%, como forma de incentivar a manutenção do interesse em avançar nas tarefas auditivas apresentadas^(24)^.

- Reavaliação

Os sujeitos do GI foram reavaliados logo após as 8 sessões do treinamento auditivo (*Momento B*), enquanto que os sujeitos do GC foram reavaliados após 3 meses do diagnóstico, sem terem recebido qualquer tipo de intervenção (*Momento C*). Por fim, após três meses do fim da intervenção (*Momento D) *os sujeitos do GI foram reavaliados pela última vez. Em todas as avaliações foi mantida a mesma bateria de testes apresentada no item D.

**Análise Estatística**

Os resultados dos obtidos foram planilhados e submetidos à análise estatística descritiva (Statistical Analysis System, versão 9.4) com apresentação de tabelas de frequências para variáveis categóricas e medidas de posição e dispersão para variáveis numéricas. Para comparação de proporções foi utilizado o teste exato de Fisher,para comparação de medidas numéricas entre 2 grupos foi aplicado o teste de Mann-Whitney. Para comparação de proporções avaliadas em 2 momentos foi aplicado o teste de McNemar. Para comparação dos resultados dos testes entre 2 momentos foi aplicado o teste de Wilcoxon para amostras relacionadas. Para comparação dos resultados dos testes entre 3 momentos foi utilizado o teste de Friedman, seguido pelo teste de Dunn para localização das diferenças, quando necessário. O nível de significância adotado para os testes estatísticos foi 5%, os dados que se enquadraram em tal condição encontram-se destacados em negrito nas tabelas.

## RESULTADOS

Os resultados foram apresentados em itens seguindo a seguinte ordem: Caracterização da Amostra, Momento A dos grupos GC e GI, Comparação entre os Momentos A e C do GC, Comparação entre os Momentos A, B e D do GI e, por fim, Análise Comparativa Qualitativa entre os Momentos A e C do GC e B e D do GI.

Na Tabela 1 apresentam-se os dados referentes à composição dos Grupos GC e GI quanto à idade, sexo e ano escolar. Descrevem-se também os dados referentes ao desempenho dos grupos no rastreio de desempenho escolar (TDE), no rastreio cognitivo (Raven) e as pontuações atingidas na aplicação do Protocolo SAB. Observa-se que não houve diferença estatisticamente significante entre os grupos, destacando-se assim homogeneidade da amostra.

**Tabela 1:** Caracterização da amostra

**Variável**		**Grupo Controle (N=10)**	**Grupo Intervenção (N=10)**	**Total (N=20)**	**p-valor**
**Sexo**	**Feminino**	5 (50,0%)	8 (80,0%)	13 (65,0%)	0,3498^2^
	**Masculino**	5 (50,0%)	2 (20,0%)	7 (35,0%)	-
**Idade** (Median (min-max))		9,10 ± 0,88	9,40 ± 1,07	9,25 ± 0,97	0,5206^1^
**Teste RAVEN**		29,20 ± 2,94	29,80 ± 2,25	29,50 ± 2,56	0,8476^1^
**Ano Escolar**	**3º ano**	4 (40,0%)	0 (0,0%)	4 (20,0%)	-
	**4º ano**	4 (40,0%)	5 (50,0%)	9 (45,0%)	-
	**5º ano**	2 (20,0%)	4 (40,0%)	6 (30,0%)	-
	**7º ano**	0 (0,0%)	1 (10,0%)	1 (5,0%)	-
**Subteste de Leitura TDE**	**Acertos de Leitura**	30,60 ± 5,06	31,50 ± 4,33	31,05 ± 4,61	0,7899^1^
	**Percentil de Leitura**	41,75 ± 32,92	30,30 ± 31,34	36,03 ± 31,83	0,2850^1^
**Subteste de Escrita TDE**	**Acertos de Escrita**	20,30 ± 9,65	23,80 ± 8,02	22,05 ± 8,82	0,4952^1^
	**Percentil de Escrita**	18,60 ± 15,26	30,00 ± 21,63	24,30 ± 19,14	0,1713^1^
**Protocolo SAB**	**Pontuação Responsável**	39,80 ± 8,80	37,90 ± 7,61	38,85 ± 8,07	0,7613^1^
	**Pontuação Criança**	38,50 ± 8,78	37,60 ± 7,92	38,05 ± 8,15	0,8794^1^

1 Baseado no Teste de Mann-Whitney;

2 Baseado no Teste de Fisher. Média ± Desvio Padrão

Na Tabela 2 descrevem-se os dados referentes a cada teste aplicado na bateria de avaliação realizada com ambos os grupos da amostra. Houve diferença estatística na comparação do desempenho de ambos os grupos apenas no Teste SSI – Condição Sinal/Ruído (-15) na orelha direita.

**Tabela 2:** Resultados dos testes de PAC no Momento A, considerando-se os Grupos Controle e Intervenção

**Testes 1ª Avaliação de PAC – Momento A**	**Grupo Controle (N=10)** (Média ± Desvio Padrão)	**Grupo Intervenção (N=10)** (Média ± Desvio Padrão)	**Total (N=20)** (Média ± Desvio Padrão)	**p-valor**
**TDD OD**	94,00 ± 5,03	93,88 ± 5,05	93,94 ± 4,90	0,9693^1^
**TDD OE**	95,50 ± 4,38	95,38 ± 2,89	95,44 ± 3,61	0,7579^1^
**TDCV – AL OD**	9,60 ± 2,41	9,00 ± 2,91	9,30 ± 2,62	0,4917^1^
**TDCV – AL OE**	8,90 ± 2,85	7,30 ± 1,77	8,10 ± 2,45	0,2036^1^
**SSI - Condição S/R (-15) OD**	52,00 ± 20,98	35,00 ± 8,50	43,50 ± 17,85	**0,0290^1^**
**SSI - Condição S/R (-15) OE**	45,00 ± 29,91	39,00 ± 13,70	42,00 ± 22,85	0,9693^1^
**TPF OD**	50,62 ± 30,49	64,65 ± 17,81	57,64 ± 25,35	0,2725^1^
**TPF OE**	48,33 ± 30,15	59,33 ± 26,36	53,83 ± 28,14	0,3250^1^
**GIN OD**	4,60 ± 1,07	4,00 ± 1,56	4,30 ± 1,34	0,3837^1^
**GIN OE**	4,50 ± 1,08	4,20 ± 1,48	4,35 ± 1,27	0,6781^1^

1 Baseado no Teste de Mann-Whitney

**Legenda:** TDD: Teste Dicótico de Dígitos, TDCV - AL: Teste Consoante Vogal – Atenção Livre, SSI: Teste de Identificação de Sentenças Sintéticas Condição Sinal Ruído(-15), TPF: Teste Padrão de Frequência, GIN: Teste Gaps in Noise, OD: Orelha Direita, OE: Orelha Esquerda

Na Tabela 3 apresentam-se os dados comparativos entre a avaliação diagnóstica do PAC (momento A) e a reavaliação após 3 meses sem intervenção (momento C), do GC. Houve diferença estatística em relação à porcentagem de acertos nos seguintes testes: TDD e TDCV na OD, TPF na OE, SSI e GIN em ambas as orelhas.

**Tabela 3:** Resultados comparativos dos testes de PAC nos Momentos A e C, considerando- se Grupo Controle

**Testes**			**N**	**Média**	**Desvio Padrão**	**Mínimo**	**Mediana**	**Máximo**	**p-valor** ^*^
**TDD**	**OD**	Momento A	10	94,0%	5,0	85,0	93,8	100,0	
		Momento C	10	99,0%	2,4	92,4	100,0	100,0	
		Diferença	10	5	4,1	0	6,2	10,0	**0,0156**
	**OE**	Momento A	10	95,5%	4,4	87,5	96,3	100,0	
		Momento C	10	88,8%	30,9	87,5	100	100,0	
		Diferença	10	-7,4	32,6	-7,5	2,5	10,0	0,5547
**TDCV**	**OD**	Momento A	10	9,6	2,4	6,0	10,5	13,0	
		Momento C	10	12,3	1,6	10,0	12,0	15,0	
		Diferença	10	3,0	2,7	-1	3,0	8,0	**0,0117**
	**OE**	Momento A	10	8,9	2,8	5,0	8,5	15,0	
		Momento C	10	7,4	2,8	1,0	8,0	11,0	
		Diferença	10	-1,5	3,7	-9,0	-0,5	2,0	0,4766
**SSI- Condição S/R (-15)**	**OD**	Momento A	10	52,0%	21,0	30,0	50,0	100,0	
		Momento C	10	67,0%	18,9	40,0	60,0	90,0	
		Diferença	10	15,0%	17,2	-10,0	20,0	40,0	**0,0273**
	**OE**	Momento A	10	45,0%	29,9	10,0	40,0	90,0	
		Momento C	10	71,0%	20,8	40,0	65,0	100,0	
		Diferença	10	26,0%	29,5	-30,0	25,0	80,0	**0,0313**
**TPF**	**OD**	Momento A	10	50,6%	30,5	13,3	43,1	100,0	
		Momento C	10	56,0%	30,4	23,3	41,6	100,0	
		Diferença	10	5,4%	5,1	-13,3	3,3	33,4	0,3789
	**OE**	Momento A	10	48,3%	30,2	16,7	36,6	100,0	
		Momento C	10	58,0%	29,5	16,7	50,0	100,0	
		Diferença	10	9,7%	10,8	-0,1	,4	33,3	**0,0313**
**GIN**	**OD**	Momento A	10	6,0 ms	2,4	2,0	5,0	10,0	
		Momento C	10	4,6 ms	1,1	2,0	5,0	6,0	
		Diferença	10	-1,4 ms	1,8	-10,0	-0,5	0,0	**0,0625**
	**OE**	Momento A	10	5,9 ms	3,5	2,0	5,0	15,0	
		Momento C	10	4,5 ms	1,1	2,0	5,0	5,0	
		Diferença	10	-1,4 ms	3,1	-10,0	-0,5	0,0	**0,0625**

* Referente ao Teste Wilcoxon para amostras relacionadas

**Legenda:** TDD: Teste Dicótico de Dígitos, TDCV - AL: Teste Consoante Vogal – Atenção Livre, SSI: Teste de Identificação de Sentenças Sintéticas Condição Sinal Ruído(-15), TPF: Teste Padrão de Frequência, GIN: Teste Gaps in Noise, OD: Orelha Direita, OE: Orelha Esquerda

Na tabela 4, descrevem-se os resultados comparativos entre os momentos A, B e D (GI). Houve diferença estatisticamente significante nos seguintes testes: TDD OD e OE – momentos AxD; TDCV OD – momentos AxB e AxD, SSI OD e OE – momentos AxB e AxD; TPF OD – momentos AxB e BxD; TPF OE – momentos AxB; GIN – momentos AxB e AxD em ambas as orelhas.

**Tabela 4:** Resultados dos testes de PAC nos Diagnóstico (Momento A), Reavaliação Pós TAAC (Momento B) e após três meses do treinamento auditivo (Momento D), considerando-se GI

**Testes**			**N**	**Média**	**Desvio Padrão**	**Mínimo**	**Mediana**	**Máximo**	**p-valor** 2	p-valor* (AxB)	**p-valor** ^*^ **(AxD)**	p-valor* (BxD)
**TDD**	**OD**	Momento A	10	93,9%	5,1	83,8	93,8	100,0				
		Momento B	10	99,0%	1,7	95,0	100,0	100,0				
		Diferença	10	5,1	5,4	-1,3	100,0	16,3	**0,0156** 1			
		Momento D^**^	10	100%	0,00	100,0						
	**OE**	Momento A	10	95,4%	2,9	92,5	95,0	100,0				
		Momento B	10	97,8%	2,2	95,0	97,5	100,0				
		Momento D	10	99,5%	1,6	95,0	100,0	100,0	**0,0005^2^**	**0,0451**	**<0,0001**	0,0842
**TDCV**	**OD**	Momento A	10	9,00	2,9	5,0	9,5	14,0				
		Momento B	10	11,0	1,9	8,0	11,5	13,0				
		Momento D	10	12,4	2,6	8,0	12,0	17,0	**0,0482**	0,3816	**0,0469**	0,0639
	**OE**	Momento A	10	7,3	1,8	4,0	8,0	10,0				
		Momento B	10	8,0	2,8	5,0	8,0	12,0				
		Momento D	10	7,6	2,1	5,0	7,5	12,0	0,2354	---	---	---
**SSI S/R (-15)**	**OD**	Momento A	10	35,0%	8,5	20,0	35,0	50,0				
		Momento B	10	71,0%	13,7	60,0	70,0	100,0				
		Momento D	10	74,0%	10,7	60,0	70,0	100,0	**<0,0001**	**<0,0001**	**<0,0001**	0,2789
	**OE**	Momento A	10	39,0%	13,7	20,0	40,0	60,0				
		Momento B	10	66,0%	9,7	60,0	60,0	90,0				
		Momento D	10	69,0%	12,9	60,0	65,0	100,0	**<0,0001**	**<0,0001**	**<0,0001**	0,8638
**TPF**	**OD**	Momento A	10	64,7%	17,8	30,0	66,7	86,6				
		Momento B	10	81,4%	13,8	60,0	83,3	100,0				
		Momento D	10	76,3%	14,8	56,6	71,7	96,7	**0,0105**	**0,0044**	0,3371	**0,0220**
	**OE**	Momento A	10	59,3%	26,4	16,7	56,6	100,0				
		Momento B	10	78,7%	11,8	63,3	76,9	100,0				
		Momento D	10	77,7%	12,1	63,3	73,5	93,3	**0,0345**	**0,0393**	0,1027	0,2419
**GIN**	**OD**	Momento A	10	5,7 ms	2,2	2,0	6,0	10,0				
		Momento B	10	4,0 ms	1,6	2,0	4,0	6,0				
		Momento D	10	4,0 ms	1,4	2,0	4,5	6,0	**0,0343**	**0,0522**	**0,0067**	1,0000
	**OE**	Momento A	10	6,5 ms	3,3	2,0	5,5	12,0				
		Momento B	10	4,2 ms	1,5	2,0	4,5	6,0				
		Momento D	10	4,2 ms	1,5	2,0	5,0	6,0	**0,1003**	**-----------**	**-----------**	**-----------**

1 Baseado no teste de Wilcoxon para amostras relacionadas

2 Baseado no teste de Friedman, seguido pelo teste de Dunn (*) para localização das diferenças

** Sem variabilidade neste tempo, não entrou na análise comparativa

**Legenda:** TDD: Teste Dicótico de Dígitos, TDCV - AL: Teste Consoante Vogal – Atenção Livre, SSI: Teste de Identificação de Sentenças Sintéticas Condição Sinal Ruído(-15), TPF: Teste Padrão de Frequência, GIN: Teste Gaps in Noise, OD: Orelha Direita, OE: Orelha Esquerda

Na Tabela 5, apresentam-se os resultados qualitativos categóricos (Normal X Alterado) considerando a avaliação e reavaliação de ambos os grupos com intervalo de 3 meses. Destaca-se que no GC observou-se que, dos 10 sujeitos, 6 mantiveram testes alterados após os 3 meses de pausa entre as avaliações. No GI, não foram observadas diferenças entre ambas as avaliações, com manutenção do desempenho observado imediatamente após o TAAC e 3 meses após o final da intervenção.

**Tabela 5:** Resultados qualitativos das avaliações de PAC, considerando-se o Grupo Controle e Intervenção

**Teste PAC**		**Grupo Controle Comparação Momentos A x C**				**Grupo Intervenção Comparação Momentos B x D**			
		**Frequência(%)**	**Alterado**	**Normal**	**Total**	**Frequência(%)**	**Alterado**	**Normal**	**Total**
**TDD**	**OD**	**Alterado**	1 - 10.0%	4 - 40%	5 - 50%	**Alterado**	0 - 0%	0 - 0%	0 - 0%
		**Normal**	0 - 0%	5- 50%	5 - 50%	**Normal**	0 - 0%	10 -100%	10-100%
	**OE**	**Alterado**	1- 10%	2- 20%	3 - 30%	**Alterado**	0 - 0%	0 - 0%	0 - 0%
		**Normal**	1-10%	6- 60%	7 - 70%	**Normal**	0 - 0%	10 -100%	10-100%
**TDCV - AL**	**OD**	**Alterado**	0-0%	1 - 10%	1 - 10%	**Alterado**	0 - 0%	0 - 0%	0 - 0%
		**Normal**	0-0%	9 - 90%	9 - 90%	**Normal**	0 - 0%	10 -100%	10-100%
	**OE**	**Alterado**	0-0%	0 - 0%	0 - 0%	**Alterado**	0 - 0%	0 - 0%	0 - 0%
		**Normal**	0-0%	10 - 100%	10-100%	**Normal**	0 - 0%	10 -100%	10-100%
**SSI Condição S/R (-15)**	**OD**	**Alterado**	3- 30%	4-40%	7- 70%	**Alterado**	10-100%	10-100%	10-100%
		**Normal**	0 - 0%	3 – 30%	3 - 30%	**Normal**	0 - 0%	10-100%	10-100%
	**OE**	**Alterado**	2 - 20%	5 - 50%	7 - 70%	**Alterado**	0 - 0%	0 - 0%	0 - 0%
		**Normal**	0 - 0%	3- 30%	3 - 30%	**Normal**	0 - 0%	10-100%	10-100%
**TPF**	**OD**	**Alterado**	6 - 60%	0 - 0%	6 - 60%	**Alterado**	0 - 0%	0 - 0%	0 - 0%
		**Normal**	0 - 0%	0 - 40%	4 - 40%	**Normal**	0 - 0%	10-100%	10 - 100%
	**OE**	**Alterado**	6 - 60%	0 - 0%	6 - 60%	**Alterado**	0 - 0%	0 - 0%	0 - 0%
		**Normal**	0 - 0%	0 - 40%	4 - 40%	**Normal**	0 - 0%	10-100%	10-100%
**GIN**	**OD**	**Alterado**	0 - 0%	6- 60%	6- 60%	**Alterado**	0 - 0%	0 - 0%	0 - 0%
		**Normal**	0 - 0%	4 - 40%	4 - 40%	**Normal**	0- 0%	10-100%	10-100%
	**OE**	**Alterado**	0 - 0%	4 - 40%	4 - 40%	**Alterado**	0 - 0%	0 - 0%	0 - 0%
		**Normal**	0 - 0%	6 - 60%	6 - 60%	**Normal**	0 - 0%	10-100%	10-100%

**Legenda:** TDD: Teste Dicótico de Dígitos, TDCV - AL: Teste Consoante Vogal – Atenção Livre, SSI: Teste de Identificação de Sentenças Sintéticas Condição Sinal Ruído(-15), TPF: Teste Padrão de Frequência, GIN: Teste Gaps in Noise, OD: Orelha Direita, OE: Orelha Esquerda

Atualmente, encontramos na literatura e na prática clínica um debate importante: até que ponto podemos atrelar a evolução das habilidades auditivas à maturação neurofisiológica ou aos ganhos atingidos após o TAAC em diferentes momentos de avaliação de um mesmo sujeito. Tal realidade suscitou nosso problema de pesquisa e sustenta nosso interesse em compreender as diferenças observadas entre cada Momento de avaliação de cada grupo.

Com relação à distribuição da amostra quanto ao sexo, idade, escolaridade e desempenho escolar observamos que os Grupos GC e GI foram considerados homogêneos (Tabela 1). Este dado é relevante pois, assim, é possível considerar que as variáveis sexo, idade, desempenho escolar e escolaridade não influenciaram nos resultados obtidos. O mesmo panorama ocorreu em relação aos dados da primeira avaliação (*Momento A*) de ambos os grupos (Tabela 2), exceto no teste SSI, na condição sinal/ruído (-15), em que o GC teve um melhor desempenho em relação ao GI.

Concentrando-se nos dados comparativos da avaliação dos *Momentos A e C* do GC (Tabela 3), houve diferença estatística entre tais momentos. A melhora do percentual de acertos foi de no máximo 26%, observada no teste SSI da OE. É relevante destacar ainda que, nem todos os testes apresentaram melhora do percentual de acertos observados: houve diferença negativa na comparação dos momentos A e C nos testes TDD e TDCV da OE. Tais sujeitos não passaram por intervenção fonoaudiológica no intervalo entre ambas avaliações, essa diferença também pode ser justificada pela neuromaturação das habilidades auditivas que atingirão sua maturidade apenas entre os 12 e 13 anos de idade^(25)^.

Concomitantemente, o melhor desempenho do grupo no Momento C também pode estar atrelado à estimulação das habilidades auditivas associada às experiências sonoras de tais sujeitos no intervalo entre a primeira e a segunda avaliação^(1)^. Este cenário também foi encontrado em uma pesquisa com crianças em fases iniciais da alfabetização que passaram por avaliação e reavaliação de PAC com 6 meses de intervalo e sem intervenção fonoaudiológica. Observou-se melhora estatisticamente significante nos testes envolvendo habilidades auditivas de localização sonora, figura-fundo para sons linguísticos e de resolução temporal^(26)^. Entretanto, é relevante destacar que, em nossa amostra de pesquisa, essa estimulação das habilidades auditivas, parte da vida cotidiana no mundo auditivo, não foi suficiente para que todos os sujeitos atingissem a normalidade de acordo com suas faixas etárias. Dos 10 sujeitos que compuseram o GC, 60% deles não atingiu os critérios de normalidade no Momento C.

Avançando para análise comparativa dos resultados do GI nos momentos A, B e D (Tabela 4), conclui-se que houve melhora das habilidades do PAC após o TAAC e manutenção destes ganhos na reavaliação após 3 meses da finalização da intervenção. Houve diferença estatisticamente significante nos testes TDD, TDCV (apenas OD), SSI, TPF e GIN. Além disso, diferentemente do observado na análise comparativa dos Momentos A e C do GC, aqui observamos que os valores das médias obtidas em cada Momento de Avaliação são crescentes (exceto no teste GIN, em que a diminuição da média reflete melhora da habilidade de resolução temporal).

Atendo-se aos valores observados nos Momentos A e B (GI), pré e pós TAAC, observamos um aumento significativo das médias que é reflexo das habilidades auditivas aprimoradas por meio do TAAC. Houve aumento das médias nos testes TDD, TDCV (OD), SSI e TPF. Além disso, avançando para a comparação das médias obtidas nos Momentos B e D, 3 meses após o final do TAAC, tais valores se mantêm e até mesmo aumentam (exceto no TPF, permanecendo porém pontuação dentro do critério de normalidade). Tais constatações fortalecem nossa hipótese de efetividade do TAAC e da manutenção das habilidades aprimoradas no contexto longitudinal de avaliação.

Outras pesquisas também reconheceram a eficácia do treinamento auditivo em crianças com dificuldades escolares^(27)^, desvios fonológicos e até mesmo com histórico de otites médias. Alguns estudos focados na análise de efetividade do TAAC em crianças com dificuldades escolares diagnosticadas com Dislexia^(28)^ e Transtorno do Déficit de Atenção e Hiperatividade (TDAH), em que o TAAC mostrou-se eficaz para a adequação das habilidades auditivas alteradas. O TAAC também mostrou-se como eficiente em crianças com histórico de alterações fonológicas submetidas a intervenção, com constatação de melhora e adequação dos processos gnósicos auditivos pós TAAC^(29)^. Em investigações relacionadas à eficácia do TAAC em escolares com quadro de otites de repetição^(10)^ os resultados destacaram que o TAAC desencadeou uma diferença estatisticamente significante para todos os testes comportamentais na comparação pré e pós TAAC, com melhor desempenho pós intervenção nos testes de ordenação temporal, resolução temporal e figura-fundo.

A eficácia do TAAC também é reconhecida em crianças com TPAC, mas sem dificuldades escolares submetidas à intervenção ^(9)^. Um estudo acompanhou 30 crianças cujos resultados de avaliação do PAC continham pelo menos dois testes alterados. Após o TAAC apenas 11 crianças mantiveram pontuações abaixo do esperado e 19 crianças atingiram pontuações de acordo com os critérios de normalidade^(8)^.

A investigação quanto à manutenção das habilidades conquistadas após a intervenção por meio do TAAC foi objeto de estudo de outras pesquisas em que foi constatada a manutenção das habilidades auditivas num caráter longitudinal. Schochat et al.^(30)^, acompanharam longitudinalmente 20 indivíduos de 8 a 24 anos que passaram por treinamento auditivo. Na reavaliação realizada, 85% dos sujeitos melhoraram com o TAAC e mantiveram tais ganhos ao longo do tempo, reforçando a eficácia do TAAC na maioria dos casos estudados. Outra pesquisa, realizada com dez indivíduos, de idades entre 7 e 14 anos, na primeira avaliação, e entre 8 e 17 anos, na última reavaliação, observou melhora das habilidades após TAAC, e a manutenção de tais resultados, mesmo após três anos do final da estimulação^(31)^.

Os testes da bateria de avaliação do PAC são considerados na literatura como testes estáveis, comprovados em estudos de tipo teste e reteste em indivíduos com desenvolvimento típico^(32)^. Sendo assim, mudanças das respostas em momentos diferentes de avaliação podem refletir modificações no sistema auditivo e em suas respectivas funções. Tais mudanças podem ser reflexo de fatores como compensações após lesões, pela maturação do próprio sistema auditivo ao longo da primeira infância ou ainda pelo aprendizado promovido por uma determinada intervenção.

No nosso estudo, considerando o curto período de intervalo entre os momentos de avaliação (*Momento A e B*– 8 semanas; *Momentos B e D* - 3 meses) e a manutenção destes resultados, mesmo após o fim da intervenção no GI, inferimos que as mudanças observadas em relação ao desempenho dos sujeitos entre cada avaliação estejam atreladas ao aprendizado promovido pelo TAAC. Essa inferência baseia-se no fato de que o intervalo de 8 semanas seria insuficiente para relacionarmos tais mudanças puramente à maturação do sistema auditivo, assim como observado no estudo de Filippini et al.^(31)^.

Na Tabela 5 observamos que no GI todos os sujeitos mantiveram resultados dentro do esperado mesmo após a finalização da intervenção fonoaudiológica por meio do TAAC. Enquanto, no GC, o intervalo de 3 meses não foi suficiente para que todos os sujeitos cujas habilidades auditivas encontravam-se alteradas atingissem o padrão de normalidade adequado à faixa etária. Seis sujeitos mantiveram pontuações abaixo do esperado, tendo sido convidadas a realizar o TAAC após a reavaliação. Tais dados sustentam a prática clínica de indicação do TAAC, visto que a maturação fisiológica das habilidades auditivas, diante de outros fatores, pode ter seu curso normal modificado e até mesmo não atingir a maturação completa. Diante dos impactos das alterações das habilidades auditivas no desenvolvimento de linguagem da criança, o TAAC visa, adequar a análise e interpretação dos estímulos auditivos, promovendo o processo de maturação do sistema nervoso auditivo central para garantir a possibilidade de melhor desempenho escolar.

Diante da influência das habilidades auditivas no desempenho escolar e no processo de aprendizado acadêmico, destaca-se a importância de que a criança tenha acesso à estimulação das habilidades auditivas no momento oportuno para que atinja seu desempenho pleno durante este processo, minimizando as dificuldades de leitura e escrita e atraso no processo de escolarização.

Reconhecemos a necessidade de que futuros estudos dedicados à investigação da eficácia e manutenção das habilidades auditivas conquistadas após o TAAC sejam delineados incluindo períodos mais longos de monitoramento da amostra pós-intervenção. Além disso, é importante que estes estudos contem com tamanho amostral mais amplo. As pesquisas futuras devem dedicar-se ao cruzamento dos dados de reavaliação do PAC não apenas com testes comportamentais, mas também eletrofisiológicos para investigar os efeitos do TAAC.

## CONCLUSÃO

A partir da análise dos resultados, concluiu-se que o TAAC foi eficaz para a reabilitação das habilidades auditivas de crianças com desempenho escolar prejudicado, bem como para a manutenção das habilidades adquiridas em reavaliação após 3 meses do fim da intervenção.
